# Single-Photon Emission Computed Tomography/Computed Tomography Image-Based Radiomics for Discriminating Vertebral Bone Metastases From Benign Bone Lesions in Patients With Tumors

**DOI:** 10.3389/fmed.2021.792581

**Published:** 2022-01-04

**Authors:** Zhicheng Jin, Fang Zhang, Yizhen Wang, Aijuan Tian, Jianan Zhang, Meiyan Chen, Jing Yu

**Affiliations:** Department of Nuclear Medicine, The Second Hospital of Dalian Medical University, Dalian, China

**Keywords:** radiomics, bone metastases, benign bone lesions, SPECT/CT, diagnosis

## Abstract

**Purpose:** The purpose of this study was to investigate the feasibility of Single-Photon Emission Computed Tomography/Computed Tomography (SPECT/CT) image-based radiomics in differentiating bone metastases from benign bone lesions in patients with tumors.

**Methods:** A total of 192 lesions from 132 patients (134 in the training group, 58 in the validation group) diagnosed with vertebral bone metastases or benign bone lesions were enrolled. All images were evaluated and diagnosed independently by two physicians with more than 20 years of diagnostic experience for qualitative classification, the images were imported into MaZda software in Bitmap (BMP) format for feature extraction. All radiomics features were selected by least absolute shrinkage and selection operator (LASSO) regression and 10-fold cross-validation algorithms after the process of normalization and correlation analysis. Based on these selected features, two models were established: The CT model and SPECT model (radiomics features were derived from CT and SPECT images, respectively). In addition, a combination model (ComModel) combined CT and SPECT features was developed in order to better evaluate the predictive performance of radiomics models. Subsequently, the diagnostic performance between each model was separately evaluated by a confusion matrix.

**Results:** There were 12, 13, and 18 features contained within the CT, SPECT, and ComModel, respectively. The constructed radiomics models based on SPECT/CT images to discriminate between bone metastases and benign bone lesions not only had high diagnostic efficacy in the training group (AUC of 0.894, 0.914, 0.951 for CT model, SPECT model, and ComModel, respectively), but also performed well in the validation group (AUC; 0.844, 0.871, 0.926). The AUC value of the human experts was 0.849 and 0.839 in the training and validation groups, respectively. Furthermore, both SPECT model and ComModel show higher classification performance than human experts in the training group (*P* = 0.021 and *P* = 0.001, respectively) and the validation group (*P* = 0.037 and *P* = 0.007, respectively). All models showed better diagnostic accuracy than human experts in the training group and the validation group.

**Conclusion:** Radiomics derived from SPECT/CT images could effectively discriminate between bone metastases and benign bone lesions. This technique may be a new non-invasive way to help prevent unnecessary delays in diagnosis and a potential contribution in disease staging and treatment planning.

## 1. Introduction

Bone metastases were a common event in cancer evolution. Studies had shown that nearly 70% of cancer patients had metastases at autopsy, and 80% of the primary tumors were a prostate, breast, and lung cancers, bone-related events associated with bone metastases which can seriously affect patients' quality of life (1). Among patients with primary tumors with bone metastases or benign bone diseases, the early diagnosis was important for individualized patient treatment as treatment options vary widely (2). Although bone biopsy was the gold standard for identifying benign and malignant lesions, it was not widely used in clinical diagnosis and treatment because of the invasive procedure. A noninvasive method to distinguish bone metastases from benign bone lesions was urgently needed.

^99*m*^Tc-labeled methylene diphosphonate (^99*m*^Tc-MDP) whole-body scan (WBS) was frequently used in patients with bone lesions and had high sensitivity but low specificity. The radioactive tracer ^99*m*^Tc-MDP was deposited in the bone by chemisorption and ion exchange, the abnormal uptake of the tracer reflected the osteogenic activity and local blood flow of the lesion (3). Single-Photon Emission Computed Tomography/Computed Tomography (SPECT/CT) combined anatomic and metabolic functions to improve the accuracy of anatomic localization of lesions and the specificity of bone imaging (4, 5). However, several researchers had indicated that bone metastases and benign bone lesions had similar imaging features, particularly for patients with already known cancer (6–8), it remained difficult to discriminate bone metastases and benign bone lesions as studies had shown that 14.3% of patients still had an equivocal diagnosis after SPECT/CT examination (9, 10). Moreover, SPECT/CT diagnosis mainly depended on physicians' personal experience, which inevitably had subjective factors, and it was difficult to quantify the intensity, uniformity, and heterogeneity of lesion distribution (11).

Radiomics convert digital images into mineable data through automated or semi-automatic and high-throughput methods. Radiomics could analyze the heterogeneity of tumors as a whole through hundreds of quantitative features and also analyze the quantitative relationship between tumor biological features and imaging features, which could construct models for tumor diagnosis, efficacy evaluation and prediction, and provide valuable references for clinical treatment of tumors (12, 13). The current research on radiomics mainly focused on CT and MRI (14–16). The pathological mechanism of bone metastasis was based on the disruption of the metabolic balance between osteoclasts and osteoblasts by the molecular action of cancer cells. In contrast, the benign bone disease showed inflammation and tissue remodeling of the periosteal cartilage tissue. Different osteoblastic and osteolytic mechanisms had the potential to cause different heterogeneity and distribution of radioactive tracer (17, 18). Furthermore, different from the anatomical information of the lesion provided by traditional imaging, SPECT/CT radiomics combined the anatomical information and metabolic information of the lesion to quantify the tumor heterogeneity, which had the potential to improve the diagnostic performance.

To the best of our knowledge, there were few studies related to bone diseases based on radiomics of SPECT/CT images. Therefore, the purpose of this study was to investigate the feasibility of SPECT/CT image-based radiomics in differentiating and improving diagnostic performance for bone metastases from benign bone lesions in patients with tumors.

## 2. Materials and Methods

### 2.1. Patients

**Figure 1:**
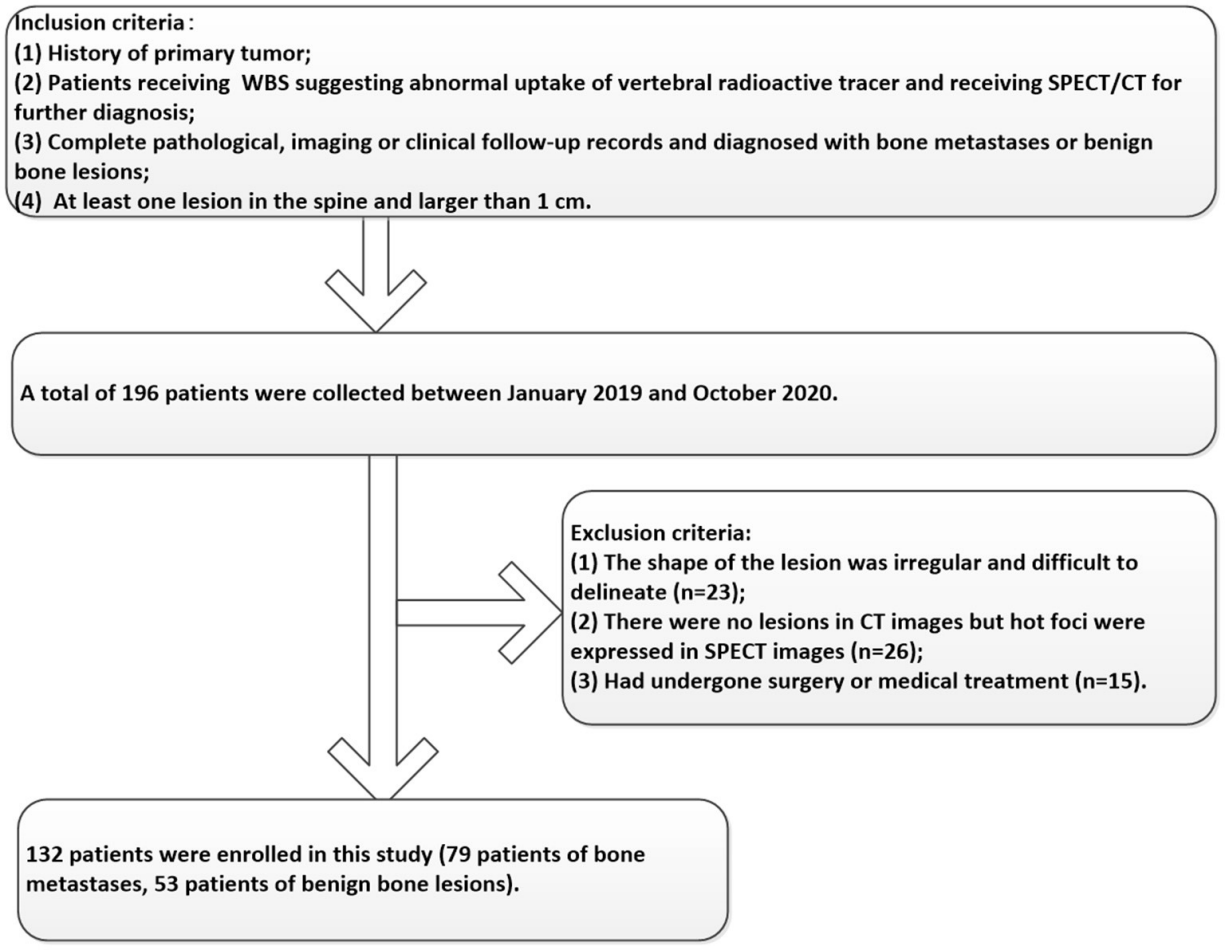
The inclusion and exclusion criteria of our study.

Participants between January 2019 and October 2020 were enrolled in this study according to the following inclusion criteria: 1) Patients a had history of the primary tumor; 2) Patients received SPECT/CT for further diagnosis because of abnormal uptake of vertebral radioactive tracer in 99mTc-MDP WBS; 3) Complete pathological, imaging, or clinical follow-up records and diagnosed with bone metastases or benign bone lesions; 4) At least one lesion in the spine and larger than 1 cm. In addition, the exclusion criteria included the following: 1) The shape of the lesion was irregular and difficult to delineate; 2) Abnormal uptake of radioactive tracer in SPECT images without lesions in CT images; 3) Had undergone surgery or medical treatment. The enrolled patients were randomly divided into the training and validation groups at the ratio of 7:3. The details of the participant's selection process were shown in Figure 1. This retrospective study was approved by the hospital ethics review committee and the requirement for informed patient consent was waived.

### 2.2. Image Acquisition

All the acquisition procedures were completed on SPECT/CT scanner equipped with a high resolution low energy parallel hole collimator (GE Healthcare Discovery NM/CT670 pro, USA). WBS was acquired within 2–5 h after intravenous administration of 15–25 mCi ^99*m*^Tc-MDP (Beijing Atomic Hi-tech Co., LTD, China), then SPECT/CT was performed immediately for further diagnosis if a suspicious lesion was found on the WBS. The SPECT acquisition parameters were as follows: double probe parallel acquisition, rotation 180 respectively, 15 s per frame, and a 128 × 128 matrix. CT scan parameters are as follows: 120 KV, 80 mA, window width of 15%, the pitch of 1.25, and slice thickness of 2.5 mm. The image reconstruction program was carried out in the XELERIS workstation (GE Medical Systems, USA), and the image fusion program was carried out in the procedure of Volumetrix MI Evolution Bone.

### 2.3. Image Analysis and Human Expert's Qualitative Classification

After summarizing all the clinical information available for diagnosis, we concluded that the diagnostic criteria for this study were based on either pathological biopsy, follow-up imaging, or progression of the clinical course. All images were evaluated and diagnosed independently by two human experts (AJT and JY) with more than 20 years of diagnostic experience for qualitative classification. The human experts made the diagnosis without being provided with clinical information but were informed that the lesion was either bone metastasis or benign bone lesion. The diagnostic results of the human experts were evaluated by weighted kappa statistics for interobserver agreement. The main criteria for the human expert's qualitative classification of bone metastases were osteolytic, osteoblastic, and mixed bone changes on SPECT/CT images and abnormal uptake of ^99*m*^Tc-MDP in the corresponding area.

### 2.4. Lesion Segmentation and Feature Extraction

All images were imported into MaZda software (version 4.6, www.eletel.p.lodz.pl) in BMP format for feature extraction, and at most two lesions were taken from each patient if the number of eligible lesions on the vertebral body was greater than three. MaZda software had been reported in previous studies to be available for radiomics image feature extraction, and it was confirmed that the radiomic features extracted by MaZda software satisfied the criteria of the Image Biomarker Standardization Initiative (IBSI) (19, 20). Before extracting features, images were normalized by using the method of μ ± 3σ (μ is the average value of the image gray value, σ is the SD of the image gray value) to reduce the influence of brightness and contrast on the gray value of the image. Two physicians (MYC and JNZ) with 5 years of diagnostic experience checked the area of abnormal uptake of ^99*m*^Tc-MDP as Region of Interest (ROI) without knowing the clear diagnosis of the lesion. The ROI was delineated on the largest cross-section of the lesion in CT and SPECT images using 2D texture sketching mode and then copied to corresponding images as needed. If the location of the lesion changes due to respiratory movement, the ROI was fine-tuned to ensure that the ROI is roughly in the same position. To ensure consistency in outlining ROI between the two physicians and to maintain stability and reproducibility of the features, 30 lesions were randomly selected for secondary outlining. Features can be divided into the following categories: gray-level histogram, gray-level absolute gradient (GrM), gray-level run-length matrix (GLRLM), gray-level co-occurrence matrix (GLCM), autoregressive model (ARM), and wavelet. Detailed information about radiomics features had been explained in previous research (21). Altogether 279 radiomics parameters were included in 6 common feature groups.

### 2.5. Feature Selection and Model Establishment

**Figure 2:**
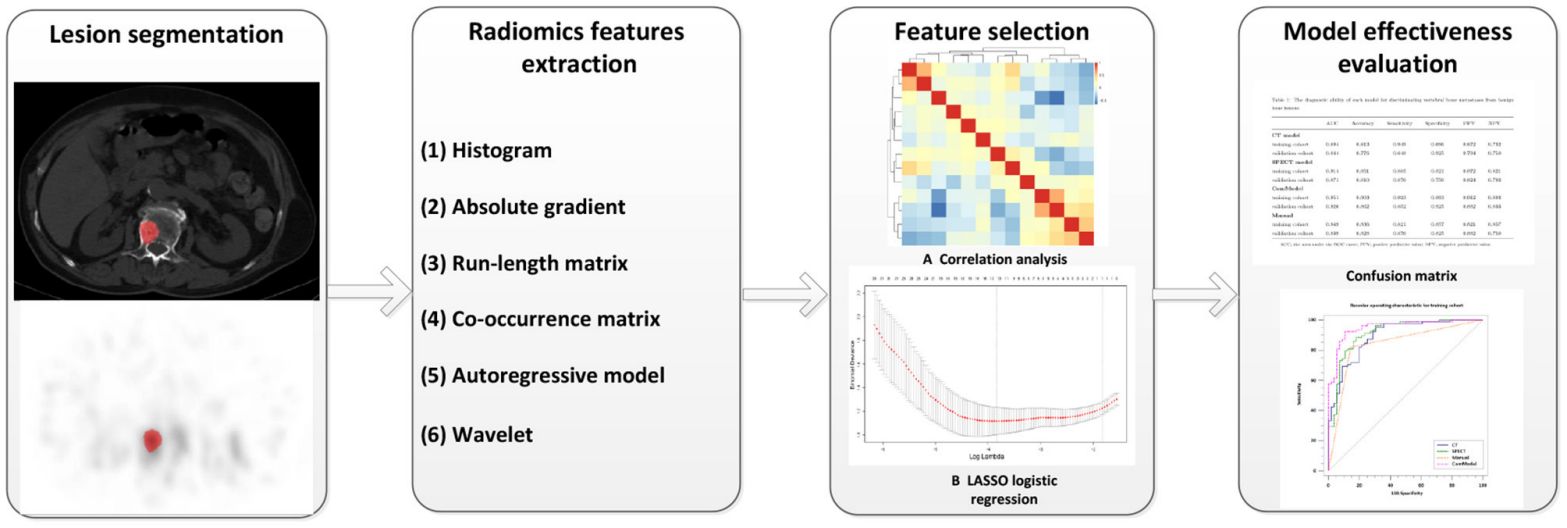
The flowchart of our study.

All features were normalized using the method of Z-score (value of feature subtract the mean value and divided by the SD) before selection. We calculate inter-texture correlation by the method of Pearson correlation and remove features with a correlation coefficient greater than 0.9 to achieve data stability and repeatability as well as to eliminate the effect of multicollinearity. In our study, if the correlation coefficient between two features is greater than 0.9, the average absolute correlation between this feature is correlation coefficient and the remaining features was compared, and the feature coefficient with the larger correlation was removed. The least absolute shrinkage and selection operator (LASSO) regression was performed on the training group for further data selection. Then the features were selected by 10-fold cross-validation based on the criteria of binomial deviance minimization. For the final selected non-zero features, we constructed a classification model by the method of multiple logistic regression. Based on these selected features, two models were established: The CT model (texture parameters were derived from CT images only) and SPECT model (texture parameters were derived from SPECT images only). In addition, a combination model (ComModel) combined with CT and SPECT features was developed in order to better evaluate the predictive value of radiomics models. The flowchart of our study was shown in Figure 2.

### 2.6. Diagnostic Efficacy of Models and Comparison

The diagnostic efficacy of all models was evaluated by the area under the curve (AUC) of the receiver operating characteristic (ROC). The confusion matrix was used to calculate the overall accuracy of the models as well as the sensitivity, specificity, negative predictive value, and positive predictive value of each model. The DeLong test was used to compare the diagnostic efficacy between each model. Calibration curves and Brier score were used to evaluate the calibration of the categorical prediction models and the good of fitness. In addition, decision curve analysis (DCA) was used to evaluate the clinical benefit of the categorical prediction models.

### 2.7. Statistical Analysis

Descriptive data were represented as the mean ± SEM. Continuous variables were compared between groups of bone metastatic and benign bone lesions with the Independent-Samples *t*-Test or the Mann Whitney *U*-test for non-normal distribution. Categorical variables between the two groups were assessed using the chi-square test or the Fisher exact test and weighted Kappa statistics were used to evaluate the interobserver agreement. All feature screening, model construction, and evaluation of the radiomics model diagnostic efficacy were performed in R software (version 4.1.1) and Python (version 3.8.1). Other statistical analyses of clinical data were performed with IBM SPSS (version 21.0) and MedCalc software (version 20.0), and *P* < 0.05 was considered as statistically significant.

## 3. Results

### 3.1. Basic Patient Information

A total of 192 lesions from 132 patients were enrolled in this study, which included 79 patients who were classified as bone metastasis (46 men, 33 women), while the remaining 53 patients were classified as benign bone lesions (32 men, 21 women). Among all of the basic clinical factors for patients in the training and validation cohorts, including gender, age, lesion form, and methods of lesions confirmation showed no significant difference between bone metastases and benign bone lesion (all *P* > 0.05). The basic information of the patient was detailed in Table 1. The primary malignancies of the 132 patients were as follows: breast cancer, *n* = 27; lung cancer, *n* = 50; prostate cancer, *n* = 25; colon cancer, *n* = 5; renal carcinoma, *n* = 5; thyroid cancer, *n* = 5; stomach cancer, *n* = 4; cervical cancer, *n* = 3; hepatocellular cancer, *n* = 3; pancreatic cancer, *n* = 2; nasopharyngeal cancer, *n* = 2; ureteral cancer, *n* = 1. The diagnosis of the 53 patients with benign lesions is as follows: degenerative lesions, *n* = 23; fractures, *n* = 15; osteoarthritis, *n* = 8; spinal tuberculosis, *n* = 7.

**Table 1:** Basic information for patients in the training and validation cohorts.

	**The training cohort**			**The validation cohort**		
	**Bone metastases**	**Benign lesions**	***P***	**Bone metastases**	**Benign lesions**	***P***
**Gender**			0.461			0.653
Female	19	10		13	11	
Male	35	26		11	7	
**Age**	59.13 ± 11.4	61.35 ± 12.32	0.508	58.46 ± 10.97	60.45 ± 11.65	0.632
Range	30–72	35–79		35–67	34–78	
**Lesion form**			0.839			0.801
Osteolytic	8	4		4	1	
Osteoblastic	53	24		23	11	
Mixed	33	12		14	5	
**Confirmed**			0.658			0.896
Biopsy	18	9		9	4	
Follow-up	76	31		32	13	

*P < 0.05 was considered to be statistically significant*.

### 3.2. Prediction Models Building and Validation

**Figure 3:**
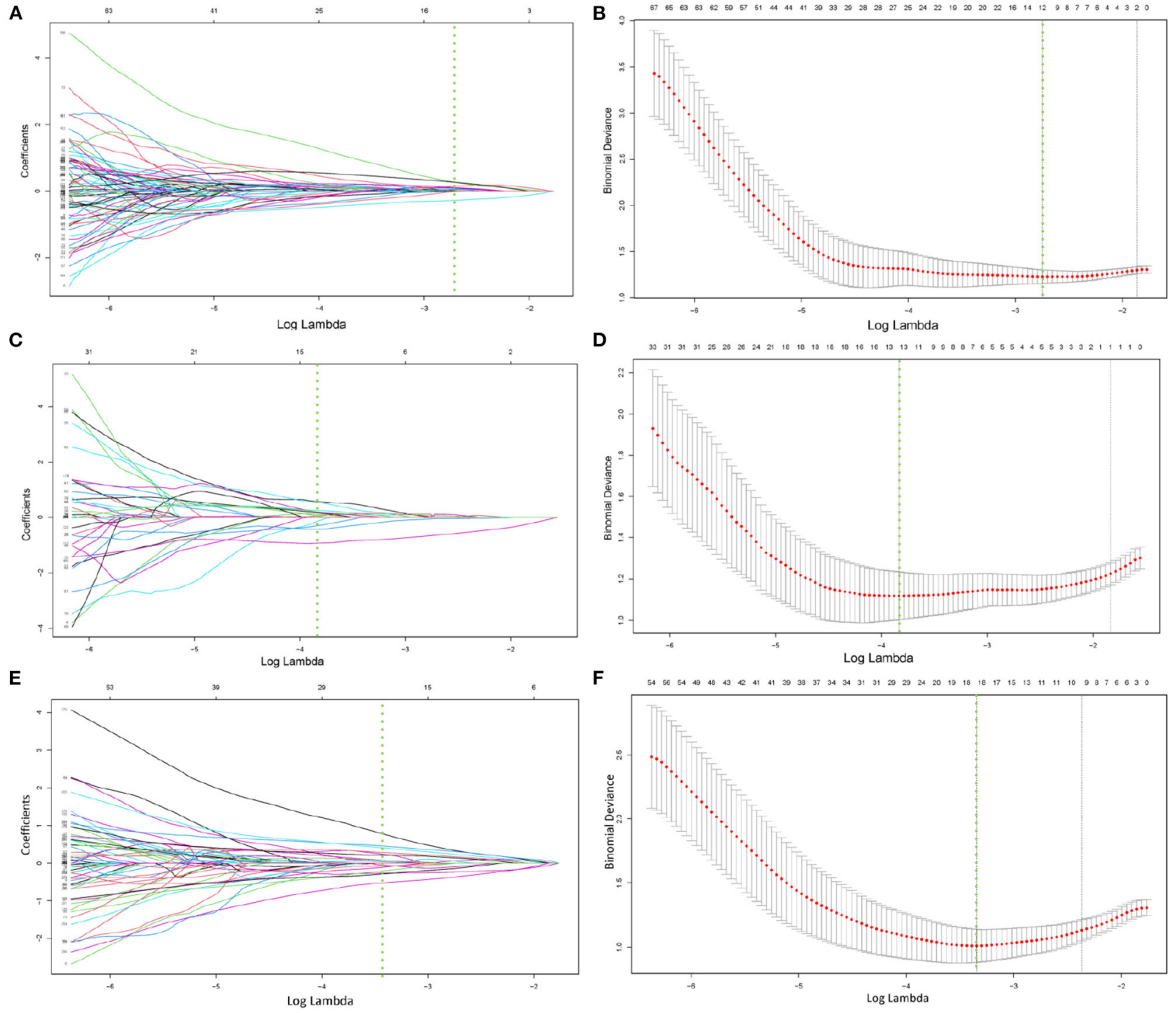
**(A–F)** demonstrated the specific process of least absolute shrinkage and selection operator (LASSO) regression analysis screening features for CT model, SPECT model, and ComModel, respectively. **(A,C,E)** showed the process of features selection. The vertical line was plotted at the optimal λ of 0.064, 0.025, and 0.035 for CT, SPECT, and ComModel, respectively. Twelve, thirteen, and eighteen factors with non-zero coefficients were finally selected for CT, SPECT, and ComModel, respectively. **(B,D,F)** showed that features selection was performed by 10-fold cross-validation with the criterion of minimum deviance.

After correlation analysis between feature groups and elimination of features with a correlation greater than 0.9, 203, and 234 features were obtained from CT and SPECT images in the training group, respectively, and then the lasso algorithm and 10-fold cross-validation were used to classify bone metastases and benign bone lesions, and finally, 12, 13, and 18 features were obtained based on CT images and SPECT images for construction of classification models, respectively, the selected features were shown in Supplementary Tables A,B. The specific process of LASSO screening features was illustrated as detailed in Figure 3. The details of the selected features obtained were demonstrated with boxplots and heatmaps in Supplementary Figures S1–S4. In the training group, the CT model, the SPECT model, and the ComModel obtained high AUC values of 0.894 (95%CI: 0.829–0.941), 0.914 (95%CI: 0.853–0.956), and 0.951 (95%CI: 0.899–0.981), respectively. ComModel have better predictive performance than CT and SPECT and there was no statistical difference between the three models after DeLong test (*P* = 0.622 between SPECT model and CT model, *P* = 0.193 between SPECT and ComModel model, *P* = 0.072 between ComModel and CT model). In the validation group, ComModel (0.926; 95% CI: 0.827–0.978) indicted better predictive performance than SPECT model (0.871; 95% CI:0.757–0.945) and significant increase than CT model (0.844; 95% CI: 0.725–0.026) (*P* = 0.063 and *P* = 0.024, respectively). In addition, SPECT model also demonstrated better predictive performance than the CT model (*P* = 0.042).

### 3.3. Diagnostic Performance Between the CT Model, SPECT Model, ComModel, and Human Experts

**Figure 4:**
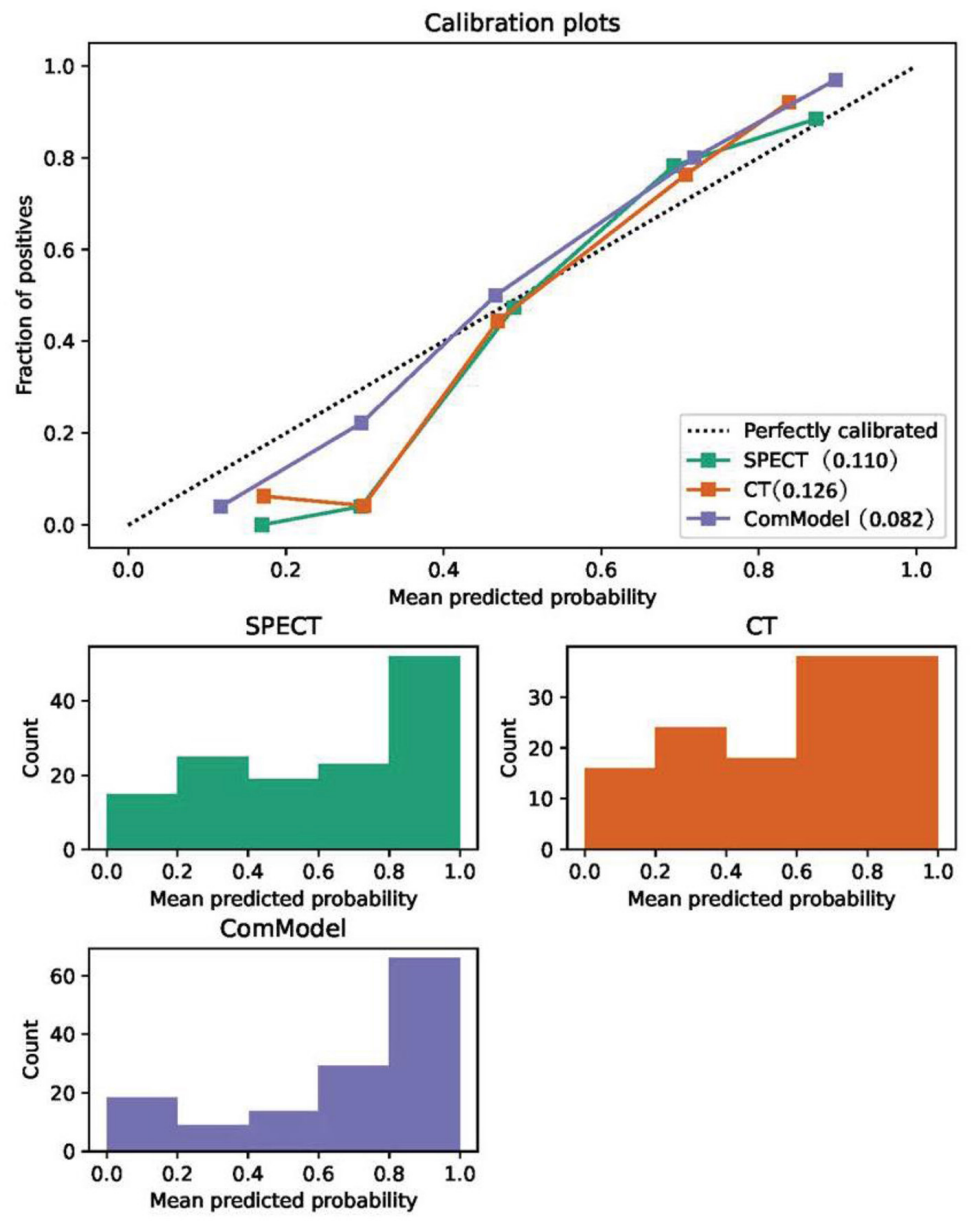
Comparison of the calibration curve and Brier score of different models. All three model's calibration curves were closed to ideal curves, indicating that the models had good fitness and predictive ability. The following figure shows the distribution of the probability of diagnosis for different models.

**Figure 5:**
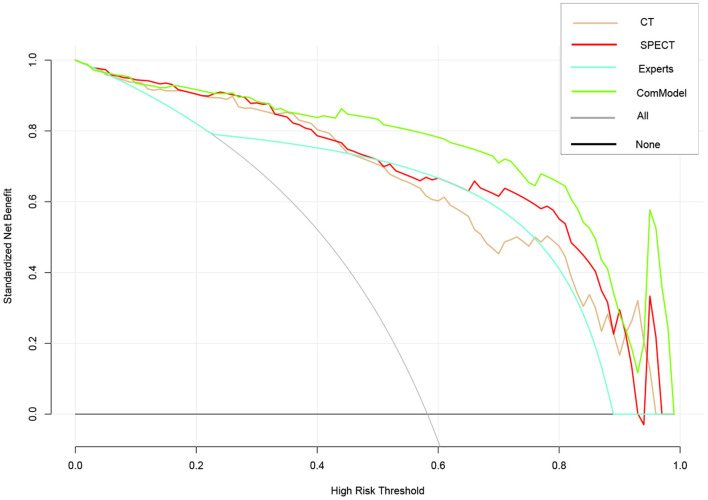
Comparison of decision curve analysis (DCA) of different models. When the threshold was 0–1, the ComModel always had a better overall net clinical gain than the other models, the SPECT model also had higher clinical gain than the human experts, and there was no significant difference between the CT model and the human experts.

**Figure 6:**
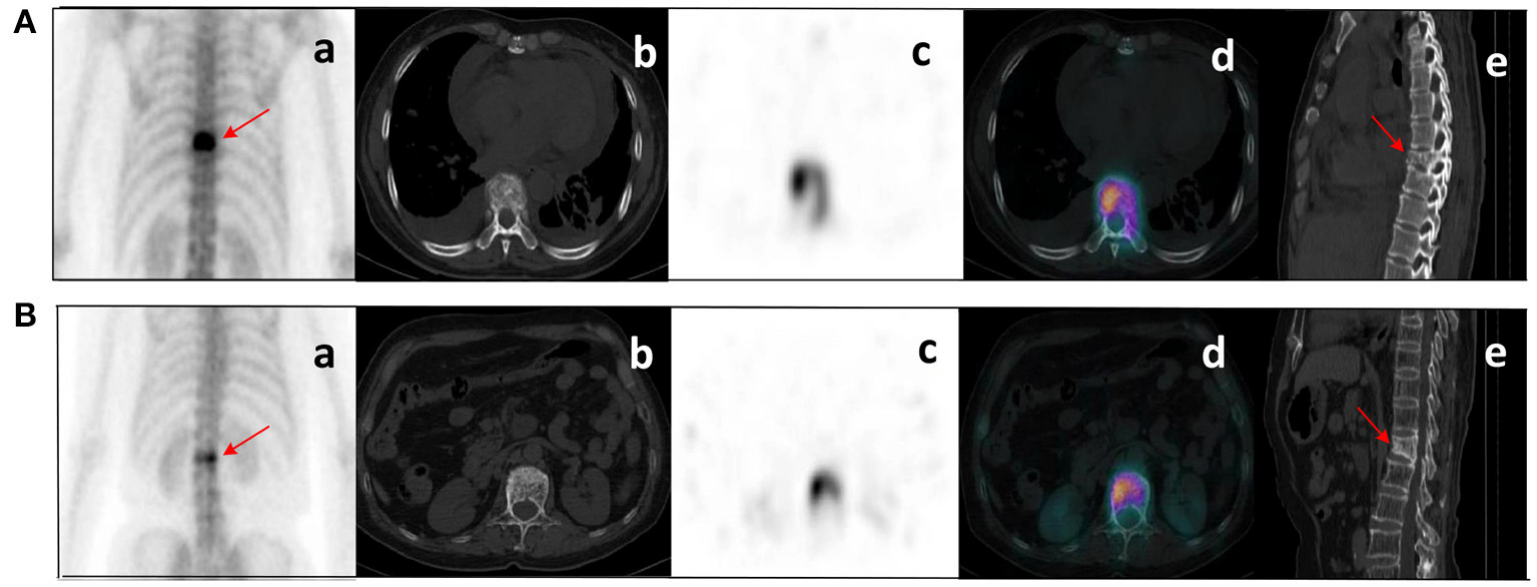
Clinical cases SPECT/CT images of bone metastases **(A)** and benign bone lesions **(B)**. The images shown are WBS image, axial CT, SPECT, fusion image, and sagittal CT (a–e, respectively). **(A)** bone metastases: a 53-year-old female with an adenocarcinoma of the left lung. Wedge-like changes of the T8 vertebral body with an abnormal concentration of radioactive tracer (arrows). **(B)** benign bone lesions: a 68-year-old female with breast cancer. Wedge-like changes of the L1 vertebral body with higher bone density and increased radioactive tracer distribution (arrows). It was difficult to determine whether lesions were metastasis with conventional images only. Lesion **(A)** was confirmed as pathological fracture due to bone metastases by pathological examination and showed systemic bone metastases at subsequent imaging follow-up. Lesion **(B)** was confirmed to be a benign compression fracture by imaging follow-up and clinical information.

**Figure 7:**
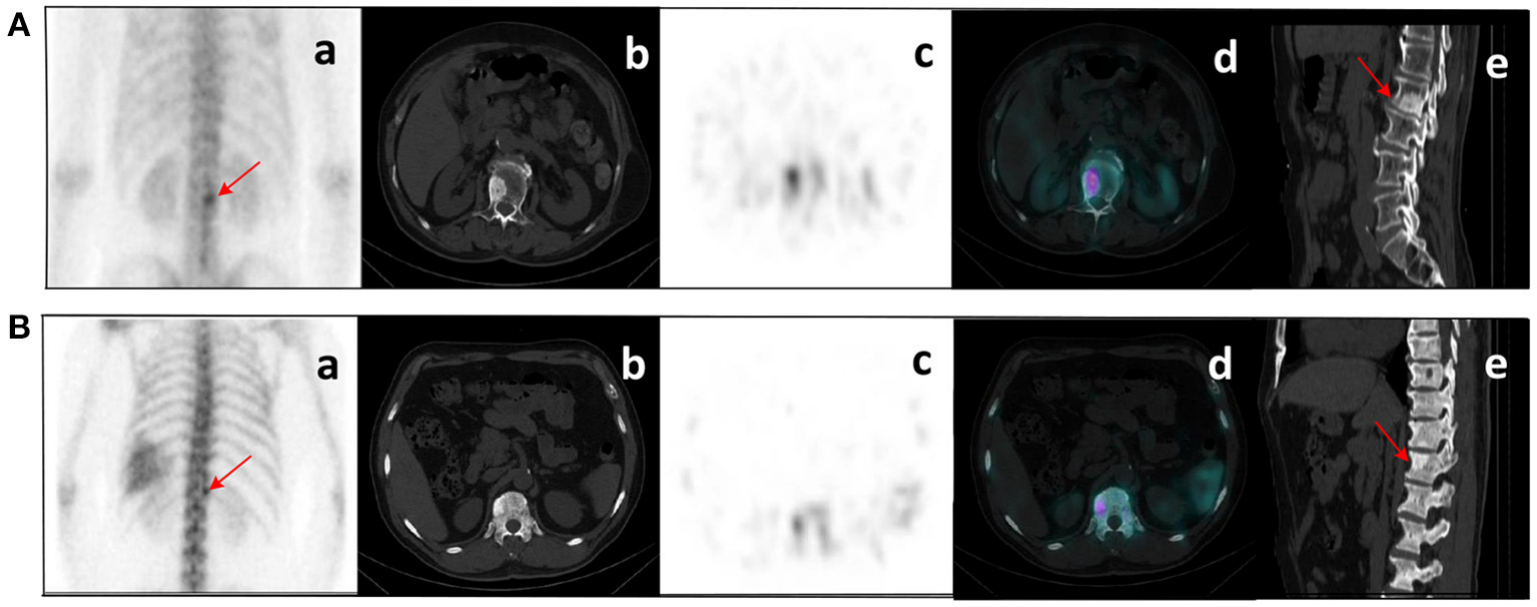
**(A)** bone metastases: a 76-year-old male with prostate cancer. Nodular high-density shadow on the right lower edge of the L2 vertebral body with a concentrated radioactive tracer (arrows). **(B)** benign bone lesions: a 60-year-old male with prostate cancer. Nodular high-density shadow on the right upper edge of the T12 vertebral body with increased radioactive tracer distribution (arrows). The cluster of radioactive tracer concentrations in the left rib(a), with fusion images suggesting bone metastasis. Lesion **(A)** showed increased concentration of tracer and increased extent of concentration with systemic bone metastases at subsequent imaging follow-up. Lesion **(B)** was confirmed not a metastasis from prostate cancer at several subsequent imaging follow-ups.

**Figure 8:**
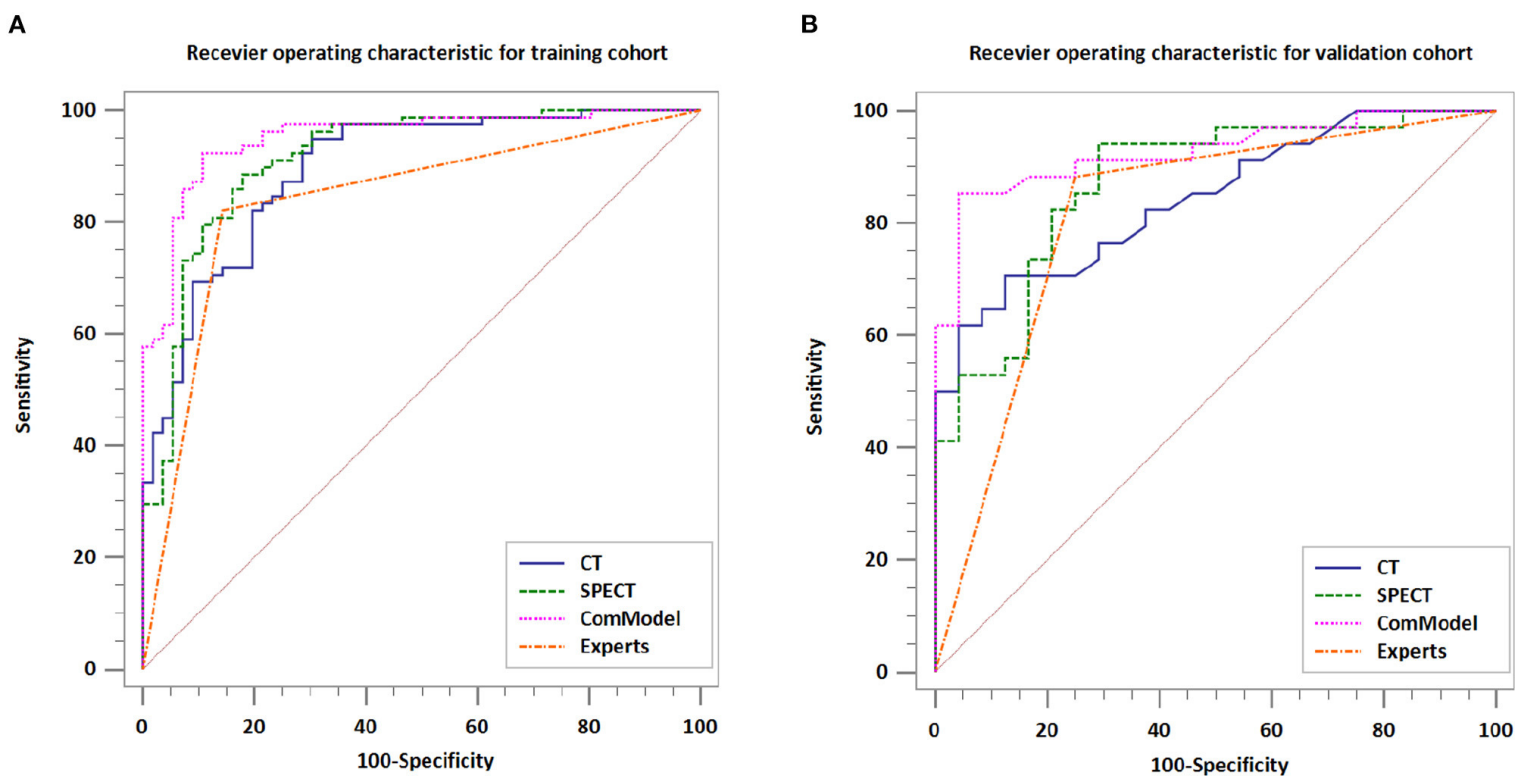
Comparison of the diagnostic performance of different models. **(A)** Receiver operating characteristic for training cohort. **(B)** Receiver operating characteristic for validation cohort.

After the Kappa test, the weighted *k*-value of the inter-observer agreement was 0.814 (95% CI: 0.713–0.895), indicating a good inter-observer agreement. The AUC value of the human experts' qualitative classification was 0.849 (95% CI: 0.775–0.907) and 0.839 (95% CI: 0.753–0.906) in the training and validation groups, respectively. In the training group, the SPECT model and the ComModel showed statistically significant differences over the human experts (*P* = 0.021 and *P* = 0.001, respectively), while the CT model showed no significant differences over the human experts (*P* = 0.091). In the validation group, the ComModel and SPECT model demonstrated greater diagnostic effectiveness over the human experts (*P* = 0.007 and *P* = 0.037 respectively), while the CT model showed no significant difference (*P* = 0.094). As for the calibration curves, all three model's curves were closed to ideal curves, indicating that the models had superior fitness and predictive ability. The calibration curve was shown in Figure 4. ComModel has a better model fitness than the CT model and SPECT model with a lower value of Brier score (0.082, 0.126, and 0.110 for ComModel, CT model and SPECT model, respectively). In the decision curves, when the threshold was 0–1, the ComModel always had a better overall net clinical gain than the other models. The SPECT model also had a slightly higher clinical gain than the human experts, and there was no significant difference between the CT model and the human experts. The decision curve was shown in Figure 5. The difficult differential diagnosis of bone metastases and benign bone lesions in clinical work was demonstrated in Figures 6, 7, respectively. A comparison of diagnostic performance between each model was shown in Table 2. The ROC curves of all models were illustrated in Figure 8.

**Table 2:** The diagnostic ability of each model for discriminating vertebral bone metastases from benign bone lesions.

	**AUC**	**Accuracy**	**Sensitivity**	**Specificity**	**PPV**	**NPV**	
**CT model**							
Training cohort	0.894	0.851	0.949	0.696	0.872	0.821	
Validation cohort	0.844	0.828	0.648	0.925	0.853	0.792	
**SPECT model**							
Training cohort	0.914	0.866	0.885	0.821	0.885	0.839	
Validation cohort	0.871	0.845	0.870	0.750	0.853	0.833	
**ComModel**							
Training cohort	0.951	0.903	0.923	0.893	0.912	0.893	
Validation cohort	0.926	0.879	0.852	0.925	0.882	0.875	
**Human experts**							
Training cohort	0.849	0.836	0.821	0.857	0.821	0.857	
Validation cohort	0.839	0.828	0.870	0.825	0.882	0.750	

*AUC, the area under the ROC curve; PPV, postive predictive value; NPV, negative predictive value*.

## 4. Discussion

In this study, we constructed and validated SPECT/CT image-based radiomics model, which achieved satisfactory classification performance and outperformed human experts' qualitative classification. Radiomics model had the potential to provide additional value distinct from CT and MRI as a noninvasive and more accessible imaging method to differentiate bone metastases from benign bone disease and reduce unnecessary invasive examinations and adjustments in treatment decisions.

Previous studies had indicated that bone metastases tend to involve the pedicle rather than the vertebral body and rarely invade the extremity bone compared to benign bone disease, which tends to affect the small intervertebral joints (22). In a study of characterization of 84 solitary lesions in the extremities, Peng et al. (23) pointed out that benign bone lesions were predominant in the proximal and distal extremity bones, whereas bone metastases were predominant in the diaphyses extremity bones, but there was no significant difference in osteoblast activity between bone metastases and benign lesions (24). Although SPECT/CT had significantly improved the diagnostic efficiency of spinal lesions and could diagnose bone metastases based on the criteria of osteolytic, osteoblastic, and mixed bone changes on SPECT/CT images and abnormal uptake of ^99*m*^Tc-MDP in the corresponding area, some benign lesions such as fractures, degenerative changes, spinal tuberculosis, and osteoarthritis can also show similar bone changes in CT and abnormal uptake of radioactive tracer, furthermore, atypical bone lesions also contributed to the challenge of differentiating between the bone metastases and benign bone disease (25). In addition, these traditional imaging features were assessed through visualization and relied on the physician's subjective evaluation and diagnostic experience, despite that lesions were not always typical in the clinical work.

Considering the limitations of traditional imaging diagnosis, the semi-quantitative analysis of bone lesions had made great progress in recent years. Kuji et al. (26) used the method of conjugate gradient reconstruction with tissue zoning, attenuation, and scatter corrections applied (CGZAS) based on WBS image to prove that SUVmax is a reliable osteoblastic biomarker for differentiating bone metastasis from degenerative changes in patients with prostate cancer. In their study, SUVmax in patients with bone metastasis was significantly higher compared with degenerative changes (40.90 ± 33.46 vs. 16.73 ± 6.74). In addition, their study also showed that SUVmax was related to bone disease progression. Le et al. (27) showed that the differential of malignant bone metastases also achieved satisfactory diagnostic performance based on the factor of PSMA-RADS rating, SUVmax, and SUVmax ratio of the lesion to blood pool by 68Ga-PSMA-11 PET/CT image. In fact, SUVmax only reflects the metabolic information of the tumor within a single pixel in the image and cannot quantify the spatial heterogeneity of the overall metabolic distribution.

Compared to traditional image assessment, radiomics was a new tool that extracted image information through high-throughput methods to provide useful information for disease typing and grading, gene localization, early treatment, and prognostic assessment; Some studies had shown that radiomics had better diagnostic performance than traditional clinical in the non-invasive classification and diagnosis of diseases. Veres et al. (28) showed that SPECT radiomics could identify microscopic lesions in the rat liver and suggested that the radiomics feature skewness could identify liver tumor lesions before they exhibit altered tissue function. Carabelli et al. (29) demonstrated that the entropy of radiomic features in SPECT myocardial perfusion imaging (MPI) could evaluate coronary vascular microcirculation noninvasively and suggested that the improvement of left ventricular functional status by liraglutide would not improve the induction of coronary microvascular dysfunction in type 2 diabetes. The study of Rahmim et al. (30) combined with radiomic analysis in the routine measurement of DAT SPECT significantly improved the diagnosis of Parkinson's outcome and believed that radiomics were expected to become an effective biomarker for Parkinson's diagnosis. However, most of these SPECT radiomics studies focus on the brain and cardiovascular aspects, and there were few studies on bone diseases.

Our research showed that the radiomics models constructed based on SPECT/CT images to discriminate between bone metastases and benign bone lesions not only had high diagnostic efficacy in the training group, with AUC of 0.894, 0.914, and 0.951 for CT model, SPECT model, and ComModel, respectively but also performed well in the validation group, with AUC of 0.844, 0.871, and 0.926 for CT model, SPECT model, and ComModel, respectively. Furthermore, both SPECT model, and ComModel showed higher classification performance than human experts, which reflected the superiority of radiomics in non-invasive classification for disease diagnosis. Another finding was that the SPECT model had better diagnostic efficacy for identifying bone metastases and benign bone lesions than the CT model. We speculated that these SPECT images represent radioactive tracer uptake and metabolic information of the lesion and can detect the lesion earlier than conventional imaging, which brings additional value to the identification of the lesion and tissue specificity. In addition, SPECT radiomics may have the potential to play a crucial role in finding the optimal dose for the targeted treatment of bone lesions with radioactive nuclides and in assessing the effectiveness of treatment.

Our study showed that the feature of entropy and correlation, which were both derived from GLCM and appeared several times in the feature extracted from CT images and SPECT images, were closely related to the identification of lesions. GLCM described the spatial relationship of pixels between features and the heterogeneity of lesions, which had been reported several times in previous studies (31, 32). Previous studies had found that the feature of entropy and correlation was related to the malignancy of lesions and helped to determine lymph node metastasis (33–35). In addition, increased heterogeneity within the images may be related to the local cellular composition, proliferation, fibrillation, angiogenesis, and necrosis of the tumor, as well as the impact of continued progressive invasion and destruction of bone metastatic disease (36, 37).

For patients considering multiple lesions, we do not automatically consider that all lesions in that patient were metastatic or benign based on biopsy or follow-up data of individual lesions, and the coexistence of bone metastases and benign bone disease in multiple lesions of the spine was relatively common in clinical work. We confirmed as metastases or benign lesions by biopsy or follow-up data for each individual lesion, and if the final diagnosis of the lesion was inconclusive, the lesion was simply eliminated, although this process took substantial time, it ensured the rigor of this study. Finally, we excluded treated patients because a proportion of patients will have flare phenomenon and osteoblastic reactions after chemotherapy or radiotherapy, which could also affect the uptake of radioactive tracer.

Our study has several limitations. First, our study had an inherent limitation with a retrospective design, thus, losing a large number of follow-up results, therefore, more standardized prospective studies are needed before the method can be used in the clinic. Second, our study is single-centered, and therefore, no external validation was performed, which may have some implications in terms of model stability. Third, detailed histopathological analysis was not always possible in each case, and we confirmed bone metastases and benign bone lesions on the basis of pathological biopsy, radiological imaging follow-up, and progression of the clinical course.

## 5. Conclusion

Radiomics models based on CT and SPECT images derived from SPECT/CT can effectively discriminate between vertebral bone metastases and benign bone disease. This technique may be a new non-invasive way to help prevent unnecessary delays in diagnosis and a potential contribution in disease staging and treatment planning.

## Data Availability Statement

The original contributions presented in the study are included in the article/Supplementary Material, further inquiries can be directed to the corresponding author/s.

## Ethics Statement

Written informed consent was obtained from the individual(s) for the publication of any potentially identifiable images or data included in this article.

## Author Contributions

ZJ and JY contributed to the conception and design of the study. ZJ and MC organized the database. FZ and YW carried out data statistics and analysis. ZJ wrote the manuscript. JY and MC revised the manuscript. All authors have read and approved the final manuscript.

## Funding

This study was supported by the Nature Science Foundation of Liaoning Province of China (No. 2019-ZD-0620).

## Conflict of Interest

The authors declare that the research was conducted in the absence of any commercial or financial relationships that could be construed as a potential conflict of interest.

## Publisher's Note

All claims expressed in this article are solely those of the authors and do not necessarily represent those of their affiliated organizations, or those of the publisher, the editors and the reviewers. Any product that may be evaluated in this article, or claim that may be made by its manufacturer, is not guaranteed or endorsed by the publisher.
