# Supplementary material for: Single-Photon Emission Computed Tomography/Computed Tomography Image-Based Radiomics for Discriminating Vertebral Bone Metastases From Benign Bone Lesions in Patients With Tumors
Source: Front Med (Lausanne). 2022 Jan 4;8:792581. doi: 10.3389/fmed.2021.792581 (PMC8764284; doi:10.3389/fmed.2021.792581)
Supplement: Supplementary file 1 [file Data_Sheet_1.docx]

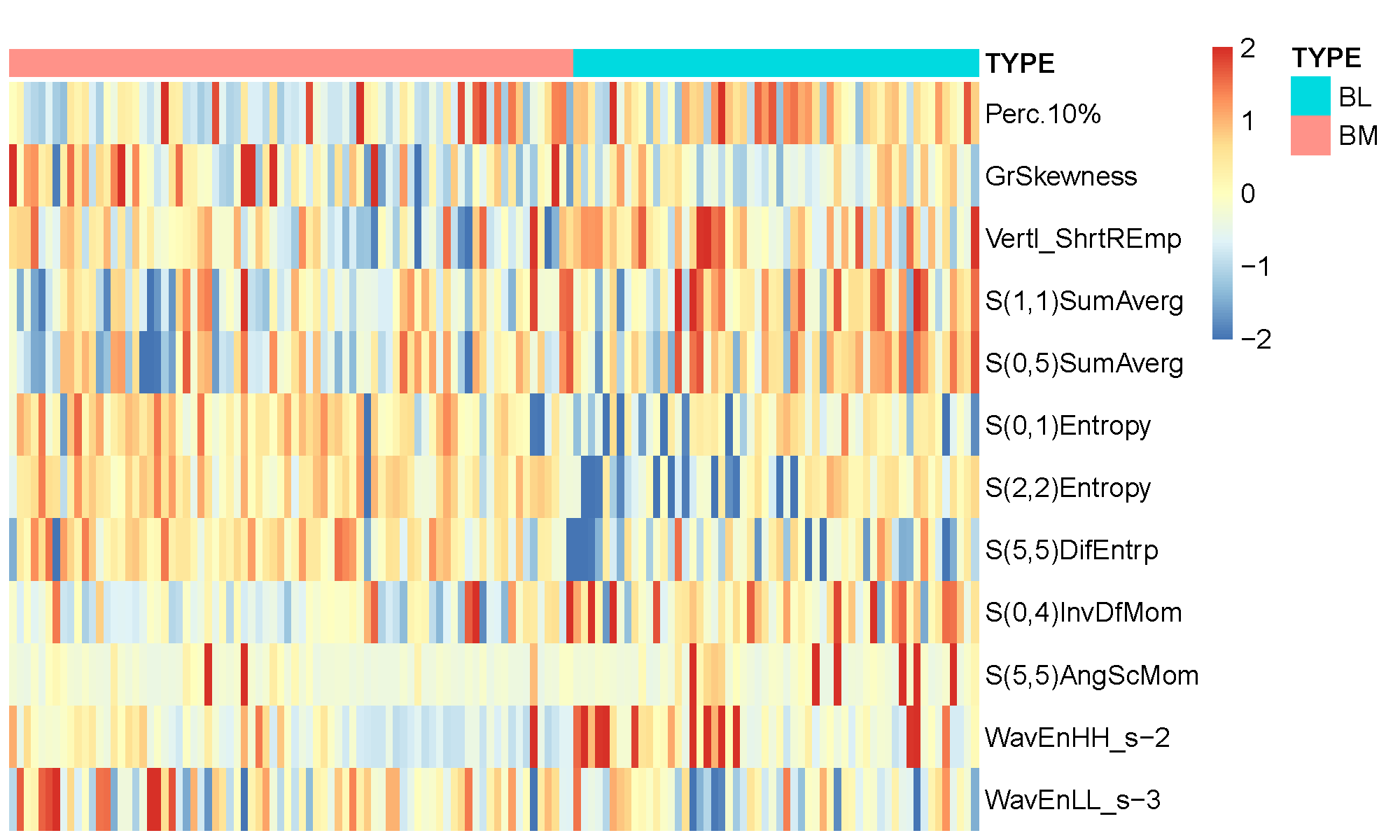


Figure S1: The heatmap of selected features for CT and SPECT model, respectively. The different shades of color represent the values of the features.


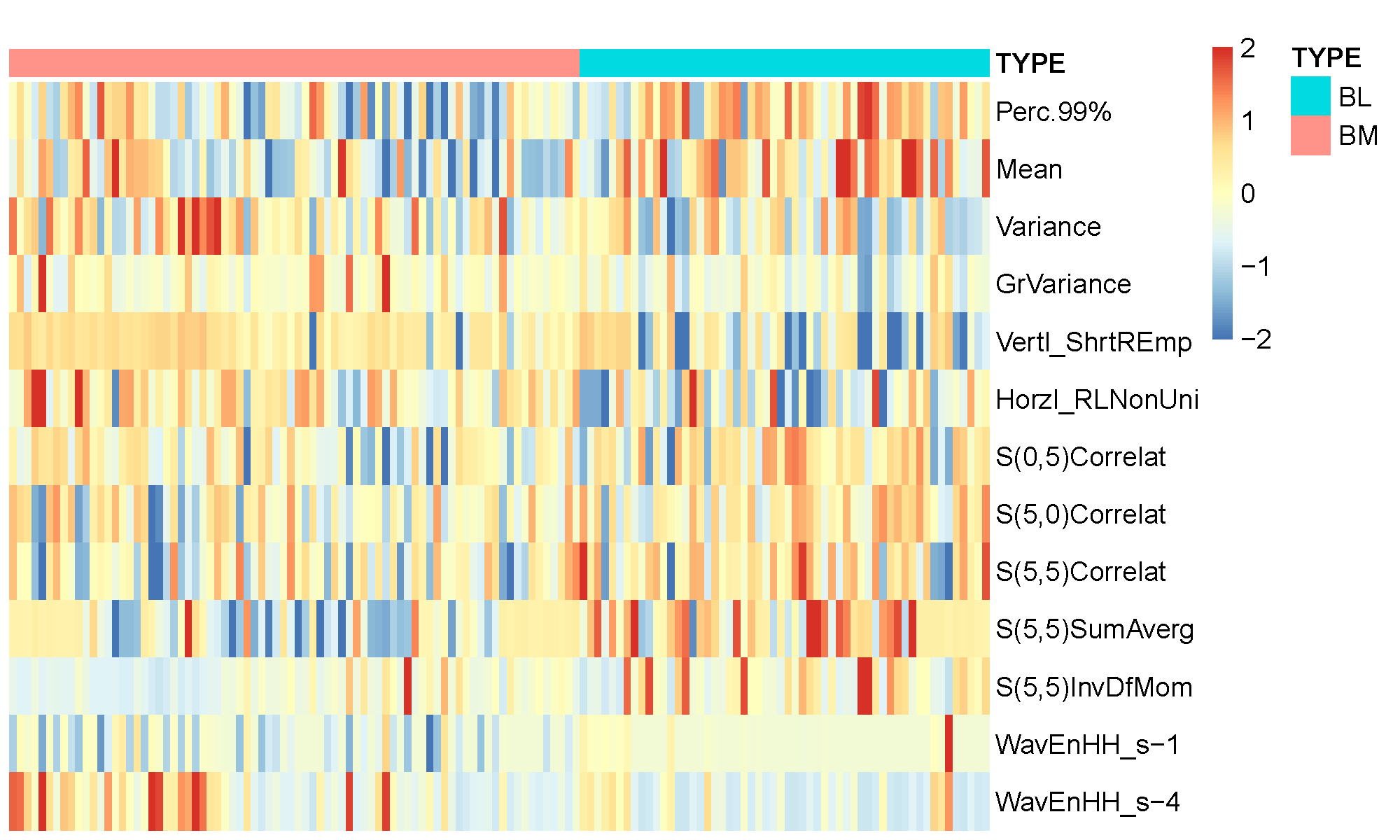


Figure S2: The heatmap of selected features SPECT model.


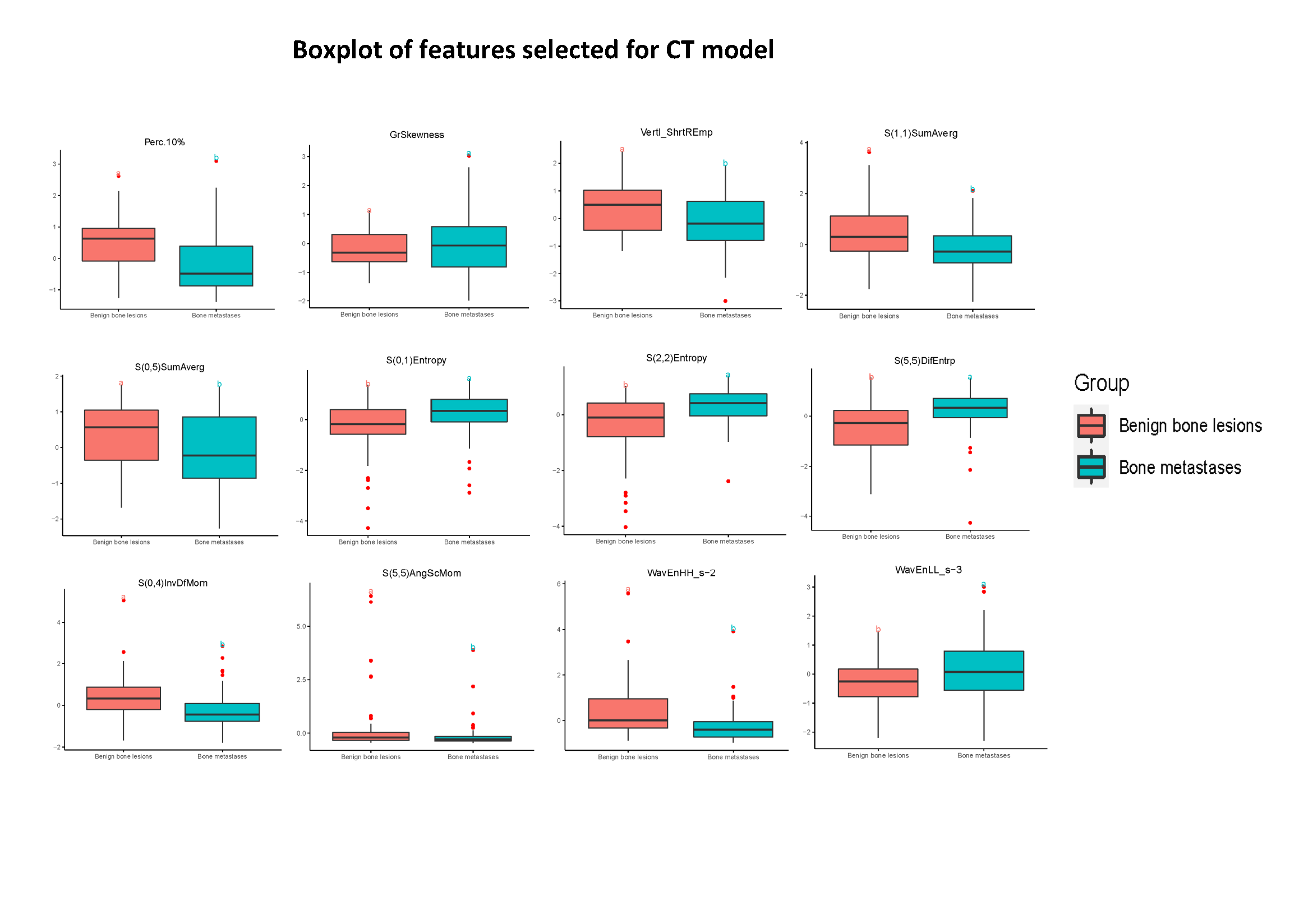
Figure S3: The boxplot of selected features for CT model. Statistically significant differences between the two groups are indicated by the letters a and b.


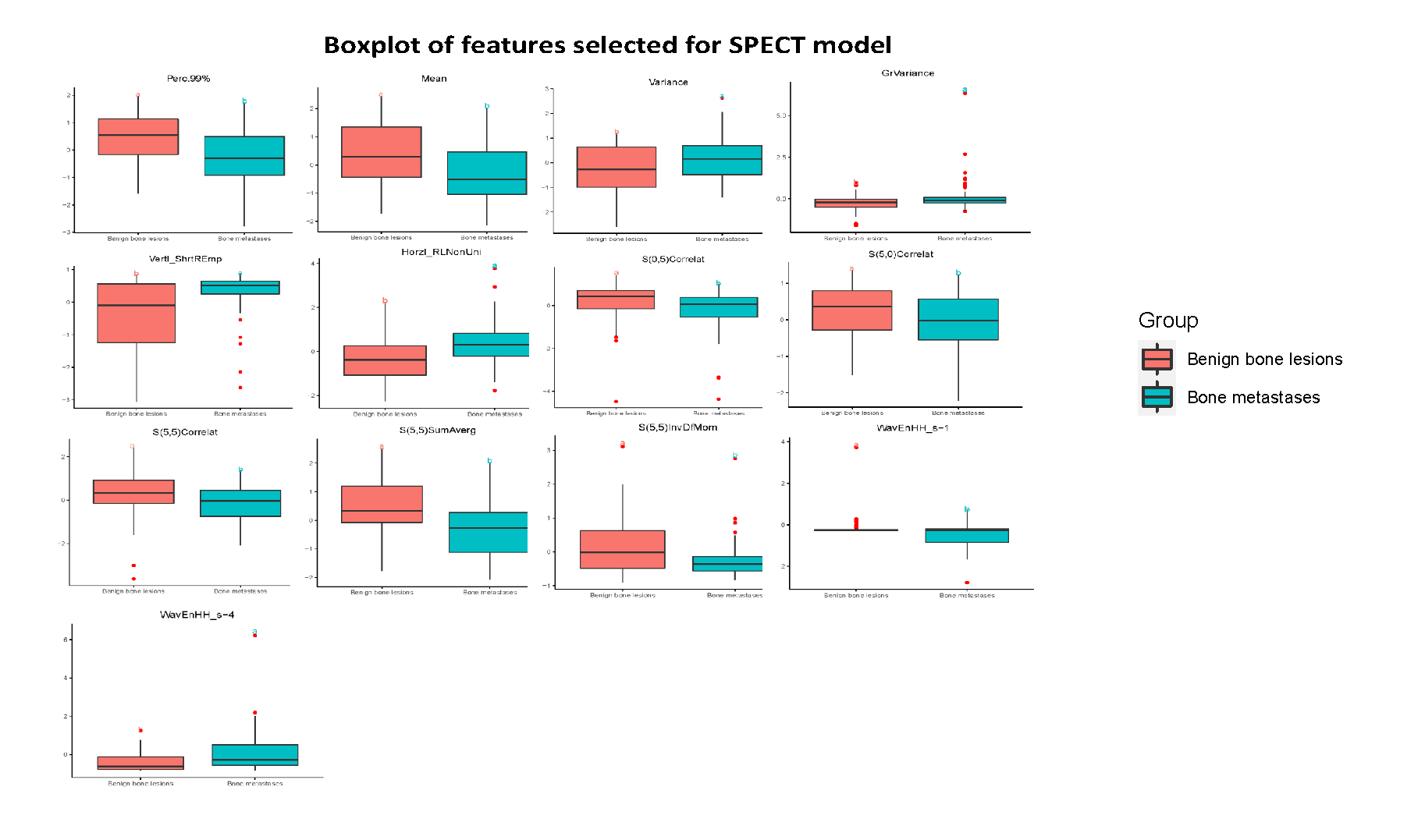


Figure S4: The boxplot of selected features for SPECT model.

Table A: Radiomics features contained in CT and SPECT models and their coefficients.

|  | **Feature** | **Coefficient** |
| --- | --- | --- |
| **CT**  **SPECT** | Intercept  Perc.10%  GrSkewness  Vertl_ShrtREmp  S(1,1)SumAverg  S(0,5)SumAverg  S(0,1)Entropy  S(2,2)Entropy  S(5,5)DifEntrp  S(0,4)InvDfMom  WavEnHH_s-2  WavEnLL_s-3  Intercept  Perc.99%  Mean  Variance  GrVariance  Vertl_ShrtREmp  Horzl_RLNonUni  S(0,5)Correlat  S(5,0)Correlat  S(5,5)Correlat  S(5,5)SumAverg  S(5,5)InvDfMom  WavEnHH_s-1  WavEnHH_s-4 | -0.691633239  0.274427609  -0.022097324  0.093327112  0.045019670  0.034585294  0.235374243  0.215472154  -0.285304181  0.109836433  0.064521909  -0.006222189  -0.698042675  0.156720003  0.023412452  0.228413466  0.077476252  -0.413216288  -0.094246227  0.133691612  0.247482402  0.484201247  -0.375621183  0.0863234522  -0.038727868  0.016042713 |

Table B. Radiomics features contained in the ComModel and their coefficients.

|  | **Feature** | **Coefficient** |
| --- | --- | --- |
| **ComModel** | Intercept  Perc.10%_CT  Vertl_ShrtREmp_CT  S(0,5)SumAverg_CT  S(0,1)Entropy_CT  S(2,2)Entropy_CT  S(5,5)DifEntrp_CT  S(0,4)InvDfMom_CT  WavEnLL_s-3_CT  Perc.99%_SPECT  Mean_SPECT  Variance_SPECT  Vertl_ShrtREmp_SPECT  Horzl_RLNonUni_SPECT  S(0,5)Correlat_SPECT  S(5,0)Correlat_SPECT  S(5,5)Correlat_SPECT  S(5,5)SumAverg_SPECT  S(5,5)InvDfMom_SPECT | -0.703487642  0.065588125  0.086070852  0.037730156  0.168750117  0.129898831  -0.262124776  0.014061316  -0.079226582  0.062465215  0.016311497  0.270849233  -0.182272312  -0.237435345  0.385837452  0.404138578  0.269276712  -0.163606798  0.043246782 |
